# Hysteresis Compensation in a Tactile Device for Arterial Pulse Reproduction

**DOI:** 10.3390/s18051631

**Published:** 2018-05-19

**Authors:** Fernando Carneiro, Paulo Abreu, Maria Teresa Restivo

**Affiliations:** LAETA—INEGI, Associated Laboratory for Energy, Transports and Aeronautics—Institute of Science and Innovation in Mechanical and Industrial Engineering, University of Porto, 4200-465 Porto, Portugal; pabreu@fe.up.pt (P.A.); trestivo@fe.up.pt (M.T.R.)

**Keywords:** hysteresis compensation, preisach model, feedforward controller, palpation simulation, tactile interface

## Abstract

This paper describes a system for training healthcare practitioners in the identification of different arterial pulses. The driving system uses a linear solenoid in an open loop force control. Due to the large hysteresis it exhibited, a form of compensation was implemented, based on the classic Preisach model of hysteresis. Implementation of said model resulted in a significant reduction of force tracking error, demonstrating the feasibility of the chosen approach for the intended application.

## 1. Introduction

Palpation of the pulse is a medical technique that allows a physician to rapidly assess the status of a given person’s cardiovascular system. It can be a good source of information, as it enables the physician to perform a rough measurement of heart rate, rhythm, strength of contraction and artery elasticity [1]. This allows estimation of the pulse waveform, which can be an indicator of the development of different cardiovascular diseases [2,3,4,5]. Examples include the pulsus bisferiens, pulsus paradoxus and pulsus alternans, that are associated with conditions such as aortic stenosis and regurgitation, hypertrophic cardiomyopathy, pericardial tamponade, restrictive pericardis and dilated cardiomyopathy [3].

Proper palpation technique requires extensive training and skill [4], and even though there are several situations for which its reliability has been confirmed [6,7] has been suggested to be unreliable under certain circumstances [8,9,10]. It has also been shown that accuracy of palpation is tied to clinical experience, training and time available [10,11,12]. Simulation plays an increasingly large role in medical training, having been applied with success to several disciplines [13,14]. The major drivers for simulation in healthcare training are patient safety and availability, as well as the lack of standard patients for training and a way to objectively assess the trainee’s performance [15,16].

Regarding simulation use in palpation, there are several examples in literature that make use of interaction, often coupled with virtual environments. These include systems for tumour detection [17,18,19,20] as well as pulse palpation [21,22,23,24,25,26].

One of the earliest commercially available simulators used for medical training was the Harvey^®^ (Laerdal, Stavanger, Norway), a mannequin that simulates several cardiopulmonary functions, including arterial pulse. This system is still used and has been shown, from very early on, to provide good results [27,28,29]. One other relevant commercial device is the Self-Teaching System (STS) for Cardiac Auscultation (Cardionics, Webster, TX, USA), that allows training of palpation and auscultation, even though no studies were found to confirm its effectiveness.

Considering the relevance of this topic and the existing solutions, this paper proposes a solution for tactile simulation of the arterial pulse based on simple, low-cost technology, that could be used for medical training. Besides enabling accurate recreation of different arterial pulses, the system was envisaged to also allow amplified reproduction of each signal, which could be beneficial in the earlier stages of learning. The main goal of the system is to be used as a training tool for current or future physicians, so that they can learn to easily distinguish between different pulse profiles. This would enable them to make early diagnoses of certain conditions using only finger palpation, without having to resort to expensive or unavailable equipment.

**Figure 1:**
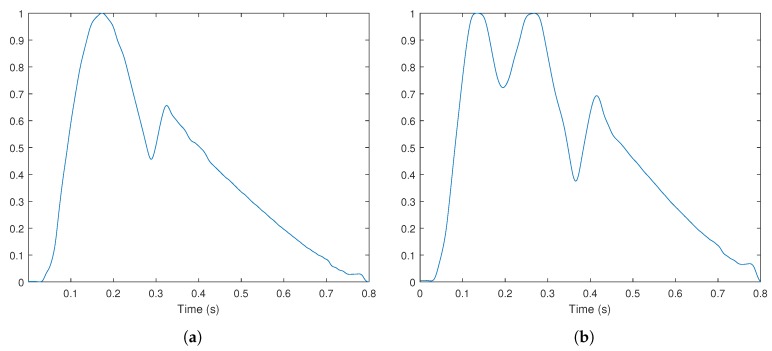
Pulse waveforms used for testing: (**a**) normal pulse; (**b**) bifid pulse. The waveforms were normalized so that a value of one represents the systolic pressure and zero the diastolic pressure.

Testing of the developed system was done using the waveforms in Figure 1. These signals are representations of arterial pulse, obtained from a waveform generator [30], and were chosen due to being two reference pulses that should be easily distinguished: normal and bifid pulse. The typical pulse waveform is characterized by a systolic pressure of 120 mmHg, a diastolic pressure of 80 mmHg and a rate of around 75 beats per minute [31].

While the proposed system might seem straightforward to implement, it posed a significant technical challenge, stemming mostly from the intention of keeping its cost low. This requirement led to a choice of actuator that proved to be affected by problems of hysteresis. Furthermore, ensuring a low cost was also incompatible with the use of a force sensor, which meant the device would have to use some form of open-loop force control.

Throughout the following sections the developed system will be detailed, with a focus on the method used to overcome the actuator hysteresis.

## 2. Proposed System

With the goal of developing a system for tactile reproduction of the arterial pulse waveform, different actuation solutions were considered. The chosen form of actuation was a linear solenoid, as it allowed the intended linear movement and enabled the production of a low-cost and compact solution. The only issue it posed was a significant hysteresis, which was surpassed through the procedure detailed in Section 3.

### 2.1. The Mechanical System

**Figure 2:**
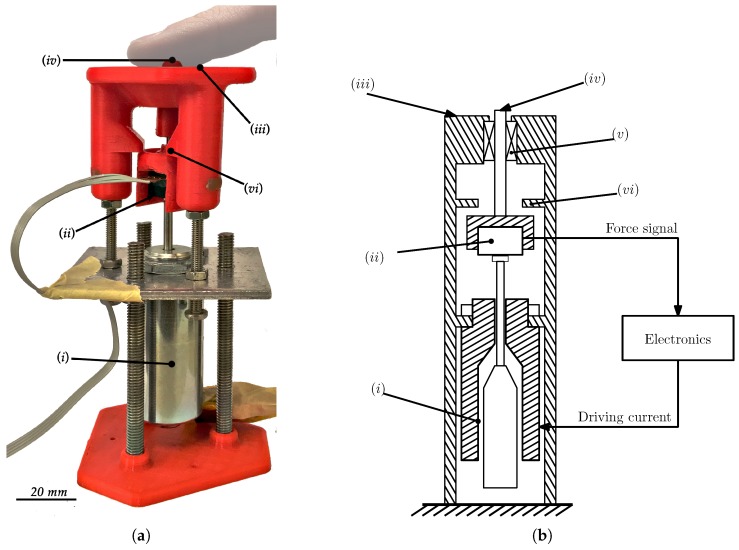
Depictions of the tested prototype: (**a**) photograph and (**b**) diagram, where: (i) solenoid; (ii) force sensor; (iii) finger rest; (iv) end effector; (v) bearing; (vi) range limiter.

The designed system consists of a linear solenoid actuator (STA® Push DC Tubular Solenoid–195207-228, Johnson Electric, Shatin, Hong Kong) , assembled verticaly on a structure. On top of its plunger, there is a force sensor (FSG-15N1A, Honeywell, Morris Plains, NJ, USA), connected to the end effector. The end effector goes through the center of the finger rest, that sits on top of the structure, and is meant for finger placement. The designed system is shown in Figure 2.

As this is a conventional tubular solenoid, the force applied by the plunger depends on its position. Furthermore, this variation is nonlinear and may lead to a locking action if the plunger reaches the maximum extension. In order to best counter this effect and find a suitable force range, the structure was designed so that the height of the finger rest relative to the solenoid can be adjusted. Also, the range of end effector is limited by the structure, so as to prevent the aforementioned locking. The end effector has an effective movement range of about 3 mm, centred around the surface of the finger rest. With the solenoid and power supply used, the maximum force is around 15 N, at the fully extended position.

### 2.2. Electronics

In order to control the force applied to the end effector according to the desired waveform, a custom electronic control system was designed.

The designed electronic control system comprises a microcontroller (dsPIC33EP64GS504, Microchip, Chandler, AZ, USA) responsible for three main tasks: communicating with the computer, gathering data from the transducers and sending command signals to the solenoid driver.

Communication is handled by the Universal Asynchronous Receiver-Transmitter (UART) module in the microcontroller, which interfaces with an external USB to UART adapter (UM230XB, FTDI, Glasgow, Scotland).

The device is also able to measure current drawn and force exerted by the actuator. For current measurement, a current transducer was materialized through a shunt resistor connected in series with the load, on the low side. The voltage drop across the resistor is amplified by a dedicated fixed gain amplifier with a gain of 50 V/V (INA199A1, Texas Instruments, Dallas, TX, USA) and filtered by a dedicated 5th-order Butterworth low-pass filter (MAX7414, Maxim Integrated, San Jose, CA, USA). The resulting signal is captured through the microcontroller’s ADC module. Force measurement was carried out using a force sensor whose signal is also amplified, using a separate instrumentation amplifier (INA326, Texas Instruments, Dallas, TX, USA), in order to match its output range to the ADC’s input range and improve signal quality. It is also acquired through the microcontroller’s ADC module.

To drive the solenoid, a custom driver circuit was built, using a MOSFET driver (MIC4420, Microchip, Chandler, AZ, USA) and a MOSFET (DMN4060SVT, Diodes Incorporated, Plano, TX, USA). In order to control the voltage supplied to the solenoid a Pulse Width Modulated (PWM) signal is sent from the microcontroller to the solenoid driver. In order to allow a high control bandwidth, a frequency of 115 kHz was chosen for this signal.

### 2.3. Control

This system pushes a plunger against the tip of a human finger to transmit the appropriate force sensation. This requires that the signal used to drive the solenoid is such that it imposes the desired trajectory for the end effector.

In order to keep the final cost of the system low, the use of a form of open loop force control would be preferable, as it would rid the device of a typically expensive component: the force sensor.

**Figure 3:**
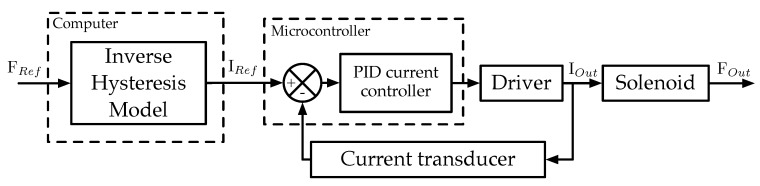
Implemented control strategy (where $F_{Ref}$ is the desired force reference, $I_{Ref}$ the current reference generated by the computer, $I_{Out}$ the current supplied to the solenoid and $F_{Out}$ the force output by the solenoid).

While the use of a fully open-loop, voltage controlled system would be ideal from an ease of implementation standpoint, initial testing revealed it to be too unreliable and unable to produce repeatable results. By switching to closed loop current control, these aspects were improved. The only major problem presented by the system was one of actuator hysteresis in the force output. As will be further detailed in Section 3, this was countered by using a control approach consisting of closed loop current control with hysteresis compensation, as depicted in the diagram of Figure 3.

Closed-loop current control was achieved by digitally implementing a Proportional–Integral–Derivative (PID) control loop on the microcontroller. Due to the fast CPU and ADC modules present on the chosen component, it was possible to implement the PID loop with a sampling frequency of 10 kHz.

In order to determine the PID control parameters, a simplified version of the system was simulated using Simulink 8.7 (MathWorks, Natick, MA, USA). The solenoid was assumed to remain static throughout the testing and its inductance and resistance were measured using an LCR meter set to the appropriate frequency. This controller was able to maintain good tracking for a great range of current levels, as well as the full range of motion of the plunger, with maximum observed error remaining under 1% of the full range.

## 3. Hysteresis Compensation

In order to counter the effects of hysteresis on the overall system response, while avoiding closed-loop force control, hysteresis compensation was applied on the actuator.

**Figure 4:**
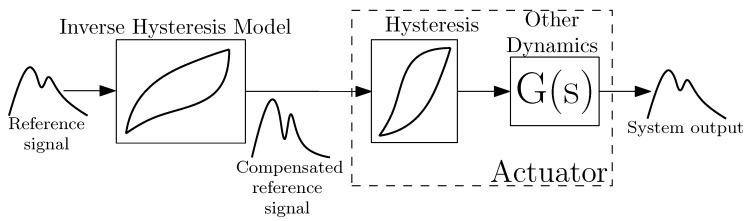
Diagram of the employed hysteresis compensation.

Hysteresis compensation involves selecting an adequate model of hysteresis, identification of the model parameters from the physical system and inversion of the model. By applying the inverse hysteresis model to the reference signal, a hysteresis compensated signal can be sent to the actuator, which should result in smaller error in the response. This effect is depicted in the diagram in Figure 4.

### 3.1. Hysteresis Model

A review of the existing literature, presented several methods for modelling the hysteresis of a physical system. There are different kinds of mathematical models for hysteresis, and they can be roughly categorized in two groups: physics-based and phenomenological-based models [32]. While physics-based models are based on the actual physical characteristics of the system, phenomenological models are devised by making use of generalized operators to model the required effect and are based on experimental data [33]. A good example of a physics-based model is the Jiles–Atherton model of ferromagnetic hysteresis [34]. Examples of phenomenological models are the Preisach model, Krasnosel’skii–Pokrovskii model and the Prandtl–Ishlinskii model [35].

**Figure 5:**
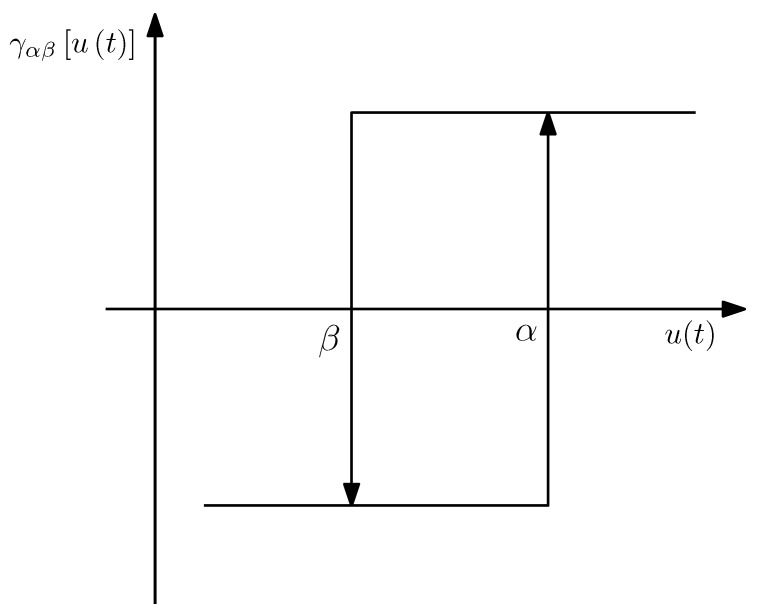
Preisach operator (hysteron).

The Preisach model is one of the most popular phenomenological models of hysteresis, making up the basis of several others. Preisach models calculate the system output as a sum of weighed basis operators. The Preisach operator, often referred to as a hysteron, can be described as a delayed relay [36] and is depicted in Figure 5.

Other Preisach-type operators include the Krasnosel’skii–Pokrovskii operator, which is a delayed relay with a final slope and the Prandtl–Ishlinskii operator, that consists of a play and stop operator [35]. For this project a model based on the Preisach operator was used, as it offered the most straightforward implementation and appeared to be compatible with the system requirements.

The Preisach model of hysteresis describes the continuous output of a system as:

(1) $$y\left(t\right)={\;\int \int \;}_{\alpha \geq \beta }\left.\mu \left(\alpha ,\beta \right)\gamma_{\alpha \beta }\left[u\left(t\right)\right]\right.d\alpha d\beta$$

In which $\gamma_{\alpha \beta }\left[u\left(t\right)\right]$ is the output of each hysteron, and $\mu \left(\alpha ,\beta \right)$, the Preisach density function, represents the weight of each hysteron. The values α and β correspond to the switching values of each relay as previously depicted in Figure 5.

**Figure 6:**
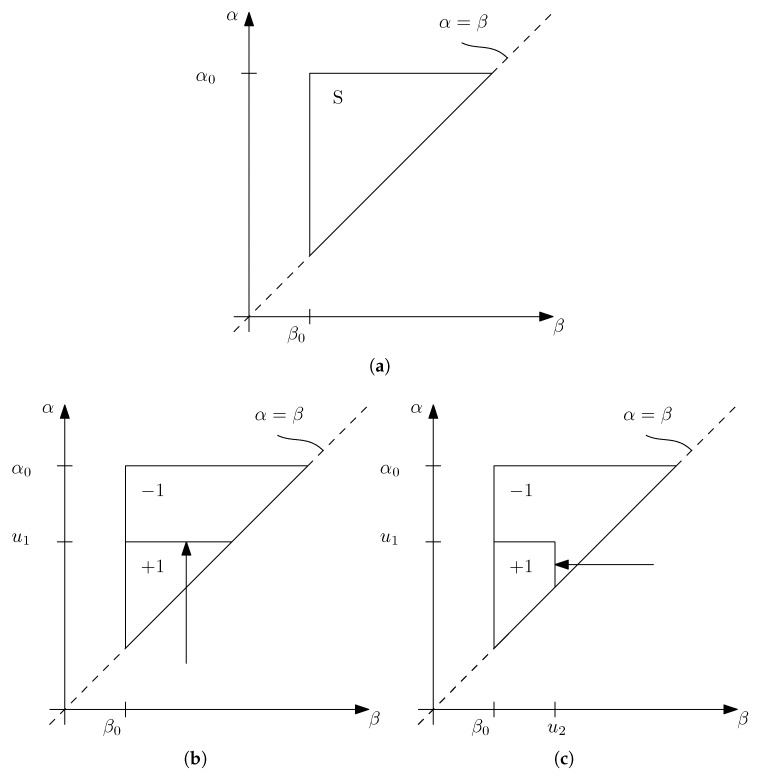
Preisach plane. (**a**) Definition of the Preisach plane; (**b**) Variation of Preisach plane values when the input is increased; (**c**) Variation of Preisach plane values when the input is decreased.

This model can also be interpreted on a geometric level, which can assist in understanding some of its properties. In Figure 6 is depicted the Preisach plane, limited by $\alpha_{0}$, $\beta_{0}$ and the line where α=β. On each point of the plane there is an individual hysteron, characterized by its switching values α and β. The output of each hysteron also depends on the system input u(t), so if the initial output is considered to be −1 and the input monotonically increases from a value inferior to $\beta_{0}$ up to $u_{1}$, some of the hysterons will be switched to +1, dividing the triangle into two regions, as depicted in Figure 6. If the input is now monotonically decreased to $u_{2}$, hysterons with β superior to $u_{2}$ will be switched back to −1 and a new line will be created. This demonstrates both the memory property and the wiping-out property of the Preisach model.

### 3.2. Identification

Implementation of this model requires identification of the density function $\mu \left(\alpha ,\beta \right)$. For this, a non-parametric method was chosen, the constrained linear least squares, as it has been proven to offer good results [37]. In this case, the constraint is that the density function is non-negative.

In order to perform this identification, the model must be discretized, as otherwise it would require differentiation of the identification (experimental) data [38]. This is done by dividing the Preisach plane in discrete intervals (cells), essentially limiting the number of hysterons that describe the system to a finite value. The output of the discrete model can be obtained by:

(2) $$y\left(t_{i}\right)=\sum_{j=1}^{n_{q}}{\hat{\mu }}_{j}A_{j}\gamma_{j}\left(t_{i}\right)+\hat{y_{0}}$$

where $n_{q}$ is the number of discrete intervals (cells) of the plane, $\hat{\mu_{j}}$ the approximate value of the weight function in each cell, $A_{j}$ the area of each cell, $\gamma_{j}\left(t_{i}\right)$ the output of each hysteron and $\hat{y_{0}}$ the bulk contribution of regions outside the Preisach plane [39].

The layout of the discrete Preisach plane for identification is done by assigning to $\alpha_{0}$ and $\beta_{0}$ the values for the extremes of the input signal, as well as dividing the α and β axes into a number of equal parts, the discretization level. The total number of cells will depend on this the discretization level, $n_{h}$, through,

(3) $$n_{q}=\frac{n_{h}\left(n_{h}+1\right)}{2}$$

Considering the input waveform has a certain number of samples throughout a limited period of time, Equation (2) can be used to build an over-determined system of linear equations:

(4) $$\left[\begin{array}{c} y\left(t_{1}\right)\\ \vdots \\ y\left(t_{n}\right) \end{array}\right]=\left[\begin{array}{ccc} A{}_{1}\gamma_{1}\left(t_{1}\right) & \cdots & A_{n_{q}}\gamma_{n_{q}}\left(t_{1}\right)\\ \vdots & \ddots & \vdots \\ A{}_{1}\gamma_{1}\left(t_{n}\right) & \cdots & A_{n_{q}}\gamma_{n_{q}}\left(t_{n}\right) \end{array}\right]\left[\begin{array}{c} {\hat{\mu }}_{1}\\ \vdots \\ {\hat{\mu }}_{n_{q}} \end{array}\right]+\hat{y_{0}}$$

In order to identify the values of $\hat{\mu }$ and $\hat{y_{0}}$, the system can be rewritten as,

(5) $$\hat{\mu }=A^{†}\left(y-\hat{y_{0}}\right)$$

where $A^{†}$ is the pseudo-inverse of the matrix in Equation (4) [40] and y the measured output of the system (in response to the identification signal). Determination of $\hat{\mu }$ and $\hat{y_{0}}$ is done through a two step procedure, which involves assigning an initial value (typically zero) to $\hat{y_{0}}$ and identifying a value for $\hat{\mu }$, which is then used to determine a new value for $\hat{y_{0}}$ and so on, until the values converge to within specified margins.

### 3.3. Implementation

Implementation of the method described above, requires the use of an identification signal that provides enough information. For this, the input should be able to stimulate every cell on the Preisach plane, which means every hysteron on the discretized plane should be individually switched on and off at least once. This is achieved by ensuring the identification signal has a number of reversals equal or greater than the discretization level of the plane.

The identification signal should also have the same range that will be expected of the model, as this will be the range for which the model will be able to predict hysteretic effects. In terms of frequency, as this is a rate-independent model, there are no specific requirements besides ensuring that it does not reach the structure’s resonant frequency.

In this case, the identification signal is a current reference, that was ensured to be properly tracked by the controller. The output value is a signal from the force sensor.

**Figure 7:**
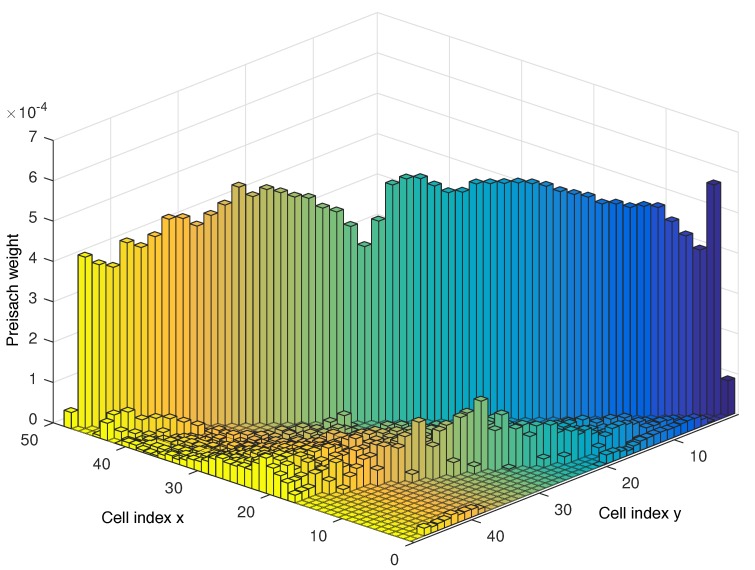
Distribution of the obtained Preisach density function.

Using this data and the procedure described in the previous section, through the algorithms detailed in [41], the Preisach density function and bulk contributions for this system were obtained, producing a model with a discretization level of 48. The obtained Preisach weights for each cell of the discretized plane, are depicted in Figure 7.

**Figure 8:**
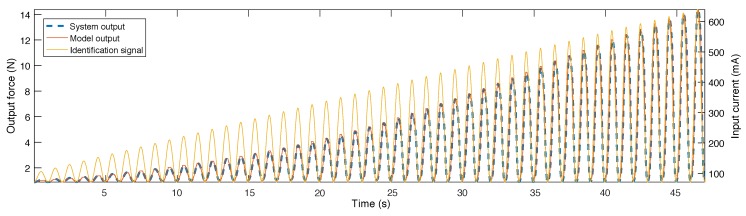
Output of the model and system for the identification signal.

After calculating the Preisach weights as well as the bulk contribution, a model was created for the system. The output of the model for the identification signal, as well as the actual output and the identification signal itself are shown in Figure 8. As can be observed, the model is able to very accurately predict the behaviour of the system for this signal.

### 3.4. Inversion

In order to apply the intended hysteresis compensation, it is necessary to invert the obtained model. The Preisach operator generally does not allow analytical inversion, as detailed in [42]. This implies that a numerical method must be selected. In this system, the closest match algorithm, proposed by [43] was used, following the implementation in [41]. This algorithm takes advantage of the discrete nature of the model, identifying an input value that generates an output as close as possible to the reference signal [40].

Although this method provides good results, the output from the model is in the form of discrete intervals, what can be problematic as it introduces undesired high frequency switching in the system. The most common way to solve this problem is by interpolating the generated signal, thereby making it continuous. In this paper, as execution time for this step was not critical, a smoothing spline was fitted to the data output by the model.

## 4. Experimental Results and Discussion

In order to evaluate the performance of the implemented hysteresis compensation, the test signals presented in Section 1 were used. These signals were scaled and offset in order to provide an output force compatible with the system. The chosen testing values were such that allowed examination of the full force range of the actuator. These values are certainly higher than what any arterial pulse is able to produce, however they can still be used in a training setting, as an amplified version for the earlier stages.

The following procedure was carried out for the two test signals:

1. The desired signal was put through the inverse model and the inverse output signal was determined;
2. The desired signal was scaled, so that its extreme values matched the extremes of the inverse signal, generating the scaled signal;
3. The inverse and scaled signals were alternately supplied to the system, the corresponding force output measured and recorded.

**Figure 9:**
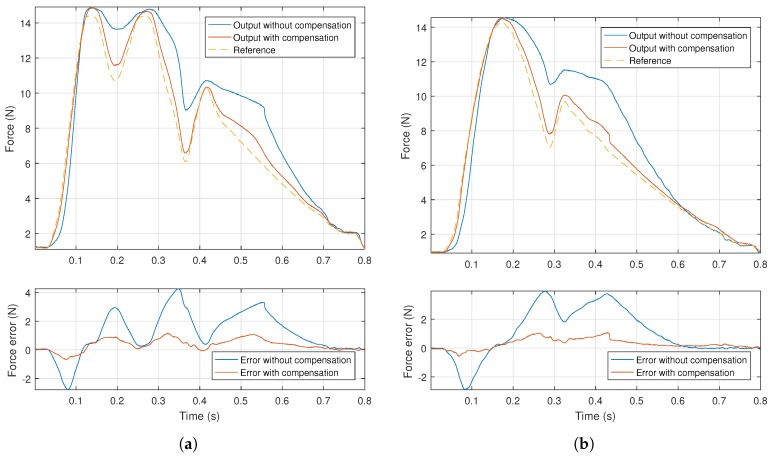
Force output by the system and error with and without hysteresis compensation, for each of the tested signals. (**a**) Bisfid pulse; (**b**) normal pulse.

This allowed direct comparison between the output waveforms, as it subjected the system to signals within the same limits, where one had hysteresis compensation, and the other did not. The results of these tests, with the waveforms superimposed are depicted in Figure 9.

As can be observed, there was noticeable decrease in error, for both of the tested signals. In the case of the normal pulse wave the average error decreased by 73.9%, while for the bisferiens pulse, the average error decreased by 70.3%. Also, maximum error relative to the full applied force was reduced, on average, from 28.7 to 7.8%.

**Figure 10:**
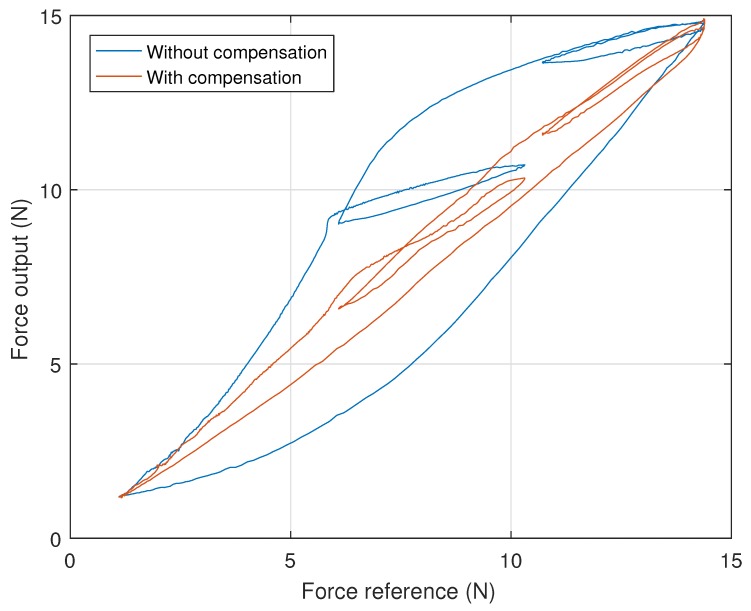
Effect of inverse compensation on the system hysteresis (input: normal pulse signal).

Furthermore, this decrease in error can be attributed to the performed hysteresis compensation, as the total system hysteresis is shown in Figure 10 to have been significantly reduced.

The remaining error can be attributed to inaccuracies in the model, effect of unmodelled dynamics, such as friction, as well as small changes in influence quantities for both the actuator and measurement system between the time of modelling and the tests. Some of the error may also be due to rate-dependent hysteresis, as the classic Preisach model cannot be used to describe rate-dependant characteristics [44].

Still, the obtained error is an acceptable starting point for the desired application and comparable to what is found in literature for similarly compensated systems [36,45,46].

## 5. Conclusions and Future Work

This paper reports the design of a system for tactile reproduction of different arterial pulses. It uses on a linear solenoid for actuation and employs an open-loop approach for force control.

The chosen actuator exhibits substantial force hysteresis. An attempt was made for its compensation by using a form of feedforward open loop control, based on the inversion of the classic Preisach model of hysteresis.

The obtained results show that the used compensation technique is effective, enabling the use of the developed system for the intended purpose of tactile waveform reproduction.

Despite the obtained results, the system can likely be improved. The mechanical system should be rebuilt in a way that exhibits less friction and using more precise and stiffer parts. The system should also be tested while interfacing with materials of different stiffness, in order to simulate interaction with human fingers.

More importantly, the system must be tested with people in order to assess its effectiveness in helping identify different pulses.
